# We May Develop Medications Effective Against COVID-19, but Can We Distribute Them Equitably?

**DOI:** 10.1089/heq.2020.0019

**Published:** 2020-07-01

**Authors:** Daniel J. Kersten, Amgad N. Makaryus

**Affiliations:** ^1^ Department of Cardiology, Nassau University Medical Center, East Meadow, New York, USA.; ^2^ New York Institute of Technology College of Osteopathic Medicine, Old Westbury, New York, USA.; ^3^ Donald and Barbara Zucker School of Medicine at Hofstra/Northwell, Hempstead, New York, USA.

There has been significant press regarding possible pharmaceutical agents with efficacy against the novel coronavirus disease 2019 (COVID-19). These medications may provide critically ill patients a greater chance of recovering from the virus. As the COVID-19 pandemic continues to escalate in the United States, concerns have been raised that persons of lower socioeconomic status, especially in urban environments, may be at greater risk of complications.^1^ These concerns are certainly valid; lower socioeconomic status is associated with a greater incidence of chronic illnesses and poorer access to health insurance and to medical care.^2–4^ Considering the risks that face underprivileged urban communities, these proposed medications could provide a vital lifeline to a particularly at-risk population. Getting these medications to this group, however, highlights additional disparities that must be addressed for there to be meaningful chance that these medications can help this at-risk population.

For one, patients of lower socioeconomic status have barriers accessing medical care in part due to limited resource availability. These medications would require a prescription, which would be difficult considering underserved urban areas have been consistently losing access to medical professionals for decades.^4^ Places with less than one physician per 3500 residents, classified by the federal government as “health professional shortage areas,” are typically areas of urban poverty.^4^ Furthermore, most of hospital closures in the past 40 years were hospitals in poor urban centers and public hospitals. The number of hospitals in U.S. major cities dropped 46% from 1970 to 2010.^4^ Even if patients obtain medical care, finding a convenient pharmacy is another challenge. Analysis of U.S. pharmacy closures found that urban pharmacies at greater risk of closing were those serving lower income communities, uninsured patients, and publicly insured patients.^5^

Whatever medication(s) are found to be useful, the demand for these treatment(s) would be massive. This will place the pharmaceutical supply chain systems under immense strain, and concerns have been raised about how demand will be met. The U.S. Food and Drug Administration has already advised that there may be potential issues with obtaining supplies for needed medical products.^6^ Demand for such life-saving medication(s) would place an even greater strain on the supply chain, thus raising prices. These increased costs would be added to the already stressed financial situations of medical systems caring for underserved urban communities.^7^ This would also affect the pharmacies in these poorer urban areas, which are more likely to struggle with maintaining adequate supplies of pharmaceuticals.^8^ This may be due to, in part, a lower density of chain pharmacies that have better resources than independent pharmacies, of which there is a greater density in poor urban areas.^8^ It is also important to consider the element of cost. The widespread demand coupled with limited supply will cause prices to rise. Persons of lower socioeconomic status are less likely to have health insurance or may have a plan that may not provide adequate medication benefits.^3,9^ Cost has already been identified as a barrier to medication compliance; and additionally, patients may adjust dosing or delay filling prescriptions due to financial worries.^9^ In a time of record unemployment and a group with limited financial security, patients may need to decide between a life-saving medication or covering other necessary expenses.

For there to be maximal benefit for any proven medication(s) against COVID-19, considerations must be undertaken to address the groups at greatest risk for complications from this virus, especially for those living in underserved communities. First, efforts must be made in these areas so that residents are able to access care, whether in hospitals or clinics. This will help not only with accessing proper care but can allow for greater screening efforts. Second, whatever medications are found to improve outcomes must be distributed to areas that have the greatest need such as a higher case burden or higher complication rates. Finally, for these medications to be used properly, efforts must be made to ensure that price protections are in place. If these conditions are not addressed, areas of urban poverty will suffer even greater consequences from COVID-19.

## Abbreviations Used

COVID-19: coronavirus disease 2019
